# Atypical Antipsychotics in the Treatment of Depressive and Psychotic Symptoms in Patients with Chronic Schizophrenia: A Naturalistic Study

**DOI:** 10.1155/2013/423205

**Published:** 2013-01-21

**Authors:** Marco Innamorati, Stefano Baratta, Cristina Di Vittorio, David Lester, Paolo Girardi, Maurizio Pompili, Mario Amore

**Affiliations:** ^1^Division of Psychiatry, Department of Neurosciences, University of Parma, Via Volturno 39/E, I-43100 Parma, Italy; ^2^The Richard Stockton College of New Jersey, Pomona, NJ 08240, USA; ^3^Mental Health and Sensory Functions, Suicide Prevention Center, Sant'Andrea Hospital, Sapienza University of Rome, Via di Grottarossa 1035, 00189 Rome, Italy; ^4^Department of Neurosciences, Ophthalmology and Genetics, Section of Psychiatry, University of Genova, Largo R. Benzi 10, 16132 Genova, Italy

## Abstract

*Objectives*. The aim of this naturalistic study was to investigate whether treatment with clozapine and other atypical antipsychotics for at least 2 years was associated with a reduction in psychotic and depressive symptoms and an improvement in chronic schizophrenia patients' awareness of their illness. *Methods*. Twenty-three adult outpatients (15 men and 8 women) treated with clozapine and 23 patients (16 men and 7 women) treated with other atypical antipsychotics were included in the study. Psychotic symptoms were evaluated using the Positive and Negative Syndrome Scale (PANSS), depressive symptoms were assessed with the Calgary Depression Scale for Schizophrenia (CDSS), and insight was assessed with the Scale to Assess Unawareness of Mental Disorder (SUMD). *Results*. The sample as a whole had a significant reduction in positive, negative, and general symptoms, whereas the reduction in depression was significant only for patients with CDSS scores of 5 and higher at the baseline. At the follow-up, patients treated with other atypical antipsychotics reported a greater reduction in depression than patients treated with clozapine, but not when limiting the analyses to those with clinically relevant depression. *Conclusions*. Atypical antipsychotics may be effective in reducing psychotic and depressive symptoms and in improving insight in patients with chronic schizophrenia, with no differences in the profiles of efficacy between compounds.

## 1. Introduction

Schizophrenia is a serious and disabling mental disorder usually associated with a decline in social and occupational functioning, and deficits in communication, control of behavior, the ability to feel pleasure, will, and initiative. The disease affects approximately 1% of the world's population, or around 1–12 adults in every 1,000, mostly in the 15–35 year-old group [1–3]. Yearly incidence is 16–40 cases per 100,000 according to the ICD-9 criteria and 7–14 cases per 100,000 using more restrictive criteria [4, 5]. The risk of developing schizophrenia during a lifetime is around 0.7% (95% confidence interval (CI): 0.3%–2.0%) [3].

The longitudinal course of schizophrenia is characterized by recurrent episodes of illness, periods of complete or partial remission, and often chronicity. Schizophrenia is linked to functional and social disability, it impacts the patients' ability to engage in productive work and social relationships, and it is associated with a reduced life expectancy as a result of accidents, high comorbidity with medical conditions, and suicide [6–17]. 

Furthermore, depression in schizophrenic patients is common and disabling [16, 18–30], while suicide is a frequent outcome in schizophrenia patients [17]. Back in the 1970s, Miles [31], in a review of 34 studies, estimated that up to 10% of schizophrenia patients die by suicide. More recently, other studies indicated a lifetime risk ranging between 2% and 5% [32, 33]. Furthermore, at least 50% of schizophrenia patients attempt suicide at least once during their lifetime.

First-generation antipsychotics (e.g., chlorpromazine and haloperidol) demonstrated efficacy for the treatment of positive symptoms [34], but their use was associated with a substantial risk of extrapyramidal symptoms and tardive dyskinesia. The introduction of atypical antipsychotics in the 1990s has had a major impact on the treatment of schizophrenia. Clozapine has demonstrated superior efficacy compared with first-generation antipsychotics and has a lower risk of causing extrapyramidal side effects, despite a significant risk of agranulocytosis [35–40]. Today clozapine is recommended when the illness has not responded adequately to previous treatment despite the sequential use of adequate doses of at least two different antipsychotic drugs, one of which is a nonclozapine second-generation antipsychotic [41]. The next set of drugs that were developed (e.g., risperidone, olanzapine, quetiapine, and aripiprazole) demonstrated at least equal efficacy and better tolerability than typical antipsychotics and improved safety compared with clozapine [34, 42–56]. Nevertheless, heterogeneity in the individual response to treatments is still the rule in schizophrenia patients [57–66], and discontinuation of treatment is a very frequent occurrence [64, 67]. Recently, Cuyún Carter et al. [68], analyzing the data from the United States Schizophrenia Care and Assessment Program, reported that only 10% of the sample had a positive outcome after two years.

Some studies have indicated that atypical antipsychotics may be effective for the depressive symptoms accompanying schizophrenia [40, 48, 69–71] and for suicide risk [72], despite some inconsistent results [73]. A few studies have also indicated that some atypical antipsychotics may increase awareness of their illness in schizophrenia patients [74, 75].

The aim of this naturalistic study was to investigate whether treatment with clozapine and other atypical antipsychotics for at least 2 years was associated with a reduction in psychotic and depressive symptoms and an improvement in awareness into their illness in patients with chronic schizophrenia.

## 2. Materials and Methods

This was a naturalistic research studying the outcome of patients with schizophrenia treated either with clozapine or other atypical antipsychotics for at least 2 years. Data collection occurred during routine outpatient visits. The data collected included patient demographics, clinical status, and suicide ideation and behaviors.

### 2.1. Participants

Twenty-three adult outpatients (15 men and 8 women) treated with clozapine and 23 patients (16 men and 7 women) treated with other atypical antipsychotics were included in the study. All the participants were patients treated at the Department of Psychiatry, University of Parma, between January 2005 and May 2006. Inclusion criteria were an age of 18 years or higher and having been treated with at least two first-generation or atypical antipsychotics with only partial response before the introduction of the current treatment. Exclusion criteria were the presence of dementia, illiteracy, and denial of informed consent.

The mean age of the patients treated with clozapine was 38.48 ± 11.45 years (Min./Max.: 20/63 years). Eighty-seven percent of them were diagnosed with schizophrenia (most with paranoid schizophrenia) and 13% with a Psychotic Disorder Not Otherwise Specified (NOS) (see Table 1). The mean age of the patients treated with other atypical antipsychotics was 45.39 ± 12.16 years (Min./Max.: 25/67 years), and 56.5% of them were diagnosed with schizophrenia and 43.5% with a Psychotic Disorder NOS.

The patients participated voluntarily in the study and gave their informed consent. The design of the research was approved by the local ethics committee.

### 2.2. Measures

Psychotic symptoms were evaluated using the Positive and Negative Syndrome Scale (PANSS) [76], depressive symptoms were assessed with the Calgary Depression Scale for Schizophrenia (CDSS) [77], and insight was assessed with the Scale to Assess Unawareness of Mental Disorder (SUMD) [78]. All rating scales were administered by psychiatrists who were expert in assessment and who were blind to the aim of the study.

Suicide attempts were defined as any self-harm act with at least some intent to die resulting in physical injuries which requested medical attention.

### 2.3. Analysis

The wilcoxon signed rank test was used to calculate the significance of changes from baseline in symptoms and insight. Mann-Whitney *U* tests were used to calculate differences between groups. Changes in outcome measures between baseline and follow-up were reported as changes in raw scores. Spearman rho correlations were reported for associations among variables. 

All the analyses were performed with the statistical package for the social science SPSS 17.0.

## 3. Results

The patients had a significant reduction in positive (*z* = −4.81; *P* < 0.001), negative (*z* = −3.17; *P* < 0.01), and general (*z* = −3.85; *P* < 0.001) symptoms. The patients also showed improvement in awareness of their illness (*z* = 4.84; *P* < 0.001) and symptom attribution (*z* = 4.79; *P* < 0.001). However, our sample of patients as a whole did not improve significantly in depressive symptoms (*z* = −0.96; *P* = 0.34), although the reduction was significant for patients with CDSS scores of 5 and higher at baseline.

The differences between the groups are listed in Table 1. At baseline, the groups differed in diagnosis (*P* < 0.05) and age at onset (*U* = 174.00; *P* < 0.05), but they did not differ for mean years spent since diagnosis (*U* = 213.50; *P* = 0.26). Furthermore, patients in the clozapine group had more positive symptoms (*U* = 156.50; *P* < 0.05) and fewer depressive symptoms (*U* = 168.50; *P* < 0.05) than patients treated with other atypical antipsychotics. The groups did not differ for number of patients with CDSS scores of 5 and higher (*P* = 0.34).

The groups did not differ in negative symptoms (*U* = 181.50; *P* = 0.07), general symptoms (*U* = 221.00; *P* = 0.34), and the SUMD awareness (*U* = 163.00; *P* = 0.10) or symptom attribution (*U* = 151.00; *P* = 0.054).

Treatment with clozapine lasted on average 5.36 ± 5.31 years (versus 4.70 ± 2.22 years for patients treated with other atypical antipsychotics; *U* = 233.50; *P* = 0.66). At the follow-up, the groups differed only in change from baseline in depressive symptoms, with patients treated with other antipsychotics reporting a greater reduction in depression (*U* = 162.50; *P* < 0.05) than patients treated with clozapine. Nevertheless, when we considered rates of response to treatment (a reduction of 50% of the baseline scores in those with a CDSS score of 5 or higher at the baseline), 71.4% of the depressed patients treated with clozapine versus 60.0% of the depressed patients treated with other antipsychotics had a good response (*P* = 0.52). Thus, despite the fact that the mean change from baseline was superior for other nonclozapine atypical antipsychotics, when we limited our analyses only to depressed patients, other antipsychotics were no longer superior to clozapine, which demonstrated nonsignificantly higher rates of remission.

The groups did not differ in the mean change in positive symptoms (*U* = 211.00; *P* = 0.24), negative symptoms (*U* = 231.00; *P* = 0.46), and general symptoms (*U* = 231.50; *P* = 0.47) as measured with the PANSS; and they did not differ in change in the SUMD awareness (*U* = 107.00; *P* = 0.29) and the SUMD symptom attribution (*U* = 135.50; *P* = 0.97).

Lastly, the change from baseline in depressive symptoms was not associated with the change in the SUMD awareness (rho = −0.15; *P* = 0.42) or symptom attribution (rho = −0.17; *P* = 0.34) dimensions. Reduction in depressive symptoms was associated only with a change in PANSS general symptoms score (rho = 0.56; *P* < 0.01), and not with changes in negative (rho = −0.15; *P* = 0.34) or positive (rho = 0.23; *P* = 0.12) symptoms. Furthermore, a reduction in depression was associated with higher scores on the PANSS negative symptoms at baseline (rho = 0.42; *P* < 0.01) and with lower CDSS scores at baseline (rho = −0.66; *P* < 0.01). Thus, a greater reduction in depressive symptoms was predicted by lower depression and higher negative symptoms at baseline, and these results did not change when we considered only patients with CDSS scores of 5 and higher.

## 4. Discussion and Conclusions

We investigated changes in psychotic and depressive symptoms and insight in patients with chronic schizophrenia treated with clozapine and other atypical antipsychotics for at least 2 years. The main finding was that atypical antipsychotics were associated with significant improvement in positive, negative, and general symptoms as measured with the PANSS, with no differences between drugs. Schizophrenia patients with clinically significant depression at baseline also had a significant reduction in depressive symptoms, which is consistent with previous studies which have indicated that atypical antipsychotics may be effective in reducing depressive symptoms accompanying schizophrenia [40, 48, 69–71].

Furthermore, the administration for long periods of time of atypical antipsychotics may be associated with an improvement in awareness into the illness, as reported by Pallanti and colleagues [74] who investigated the influence of treatment with first-generation versus atypical antipsychotics in relation to awareness into the illness and cognitive functions. The authors found that clozapine was effective in improving awareness into the illness in a group of schizophrenia patients who were shifted to clozapine treatment either because of relapse on or intolerance to conventional antipsychotics. In our sample, we did not find differences in awareness in patients given clozapine versus other atypical antipsychotics.

In our sample, patients treated with other atypical antipsychotics showed a greater reduction in depressive symptoms than patients treated with clozapine, but not when limiting the analyses to patients with clinically significant levels of depression at baseline. A reduction in depressive symptoms was associated with improvement in insight, but not with improvement in negative and positive symptoms. Furthermore, a greater reduction in depression was predicted by lower depressive and higher negative symptoms at baseline.

Our study has several limitations. First, our sample was small, and the lack of differences between groups in some analyses could have been associated with the small number of subjects we enrolled in the study. For example, we should have enrolled 281 subjects for both samples to detect a statistically significant difference between groups for the change in the PANSS positive symptoms with an alpha level of 5% and a statistical power of 80%. Second, we did not consider important outcome measures such as medication side effects and the discontinuation rate which have been reported to be very frequent in schizophrenia patients [64, 67] and which vary between drugs [67]. Furthermore, the treatment was not blind and the patients were not randomly assigned to medication. Thus, our results may be limited by a selection bias affecting the groups of patients differently. Third, we did not exclude from the sample patients with antipsychotic poly-therapy. Fourth, we did not include in the sample patients treated with first-generation antipsychotics, which limits the ability to understand whether atypical compounds have greater effectiveness than conventional neuroleptics. Fifth, the observational nature of the study is associated with the lack of placebo-treated controls, leaving the possibility that atypical antipsychotics may be not superior to placebo. Sixth, the fact that we did not use an intention to treat analysis to examine the effect of dropouts could explain the general improvement from baseline to follow-up in symptoms and awareness of illness of our patients.

In conclusions, atypical antipsychotics may be effective in reducing psychotic and depressive symptoms and in improving insight in patients with chronic schizophrenia, with no differences in the profiles of efficacy between compounds. Greater reduction in depressive symptoms is predicted by greater negative symptom severity and fewer depressive symptoms at baseline, but is not associated with improvement in negative and positive symptoms or in insight.

## Figures and Tables

**Table 1:** Differences between groups.

	Clozapine (*N* = 23)	Other antipsychotics (*N* = 23)	Test	*P*<
Characteristics at the baseline				
Age (M ± SD)	38.48 ± 11.45	45.39 ± 12.16	*U* = 182.00	0.07
Men (%)	65.2	69.6		0.50
Diagnosis (%)				**0.05**
Schizophrenia	87.0	56.5		
Psychotic Disorder NOS	13.0	43.5		
Age at onset (M ± SD)	22.91 ± 6.88	26.22 ± 7.20	*U* = 174.00	**0.05**
Years since diagnosis (M ± SD)	15.17 ± 8.11	18.57 ± 10.74	*U* = 213.50	0.26
Risperidone (%)	—	19.6	—	—
Olanzapine (%)	—	19.6	—	—
Quetiapine (%)	—	6.5	—	—
Aripiprazole (%)	—	2.2	—	—
Psychotic episodes ≥ 5 (%)	43.5	30.4		0.27
Schizophrenia in the family members (%)	13.0	26.1		0.23
PANSS positive symptoms at the baseline (M ± SD)	22.87 ± 6.82	17.83 ± 6.83	*U* = 156.50	**0.05**
PANSS negative symptoms at the baseline (M ± SD)	30.65 ± 9.24	24.70 ± 9.72	*U* = 181.50	0.07
PANSS general symptoms at the baseline (M ± SD)	48.61 ± 13.10	44.96 ± 12.02	*U* = 221.00	0.34
CDSS at the baseline (M ± SD)	2.78 ± 4.02	4.50 ± 3.85	*U* = 168.50	**0.05**
CDSS ≥ 5 (%)	30.4	40.9		0.34
SUMD awareness (M ± SD)	21.91 ± 11.24	16.95 ± 10.27	*U* = 163.00	0.10
SUMD symptoms attribution (M ± SD)	23.74 ± 11.14	17.05 ± 11.32	*U* = 151.00	0.054
Characteristics at the follow-up				
Clozapine doses (mg (range)) (M ± SD)	410.87 ± 127.88 (200/750)	—	—	—
Years of treatment with the current antipsychotic (M ± SD)	5.36 ± 5.31	4.70 ± 2.22	*U* = 233.50	0.66
Suicidal ideation (%)	26.1	21.7		0.50
Suicide attempts (%)	13.0	17.4		0.50
Change from baseline in the PANSS positive symptoms (M ± SD)	−8.43 ± 8.88	−6.70 ± 7.56	*U* = 211.00	0.24
Change from baseline in the PANSS negative symptoms (M ± SD)	−4.78 ± 11.07	−6.87 ± 10.57	231.00	0.46
Change from baseline in the PANSS general symptoms (M ± SD)	−8.96 ± 17.23	−13.39 ± 17.32	*U* = 231.50	0.47
Change from baseline in the CDSS (M ± SD)	0.48 ± 5.29	−2.05 ± 4.50	*U* = 162.50	**0.05**
CDSS response* (%)	71.4	60.0		0.52
Change in the SUMD awareness (M ± SD)	−6.71 ± 6.85	−7.92 ± 4.96	*U* = 107.00	0.29
Change from baseline in the SUMD symptoms attribution (M ± SD)	−7.81 ± 7.41	−6.62 ± 3.93	*U* = 135.50	0.97

*Response had been defined as a 50% change of baseline scores for patients with a CDSS score of 5 and higher at the baseline.
